# Supplementary material for: Validation of a Digital Headache Calendar at a Tertiary Referral Center
Source: Diagnostics (Basel). 2023 Dec 22;14(1):21. doi: 10.3390/diagnostics14010021 (PMC10795797; doi:10.3390/diagnostics14010021)
Supplement: Supplementary file 1 [file diagnostics-14-00021-s001.zip › diagnostics-2769203-supplementary.pdf]

Supplementary Figure S1. Diagnostic headache diary

DIAGNOSTIC HEADACHE DIARY

Hovedpinec

Complete the diary every evening. Tick the boxes that fit the questions best. Read the instructions carefully.

Name: ______________________ Birth Date: ______________

From date: - 20 to date: - 20

1.	Year:	Date:	M /	T /	W /	T /	F /	S /	S /
2.	Did you have a headache today<br>When did the headache begin?	no:<br>yes:<br>hour:							
3.	When did the headache disappear?	hour:							
4.	**Just before** the headache began, was there any	visual disturbance:<br>sensory disturbance:<br>difficulty speaking:	☐<br>☐<br>☐	☐<br>☐<br>☐	☐<br>☐<br>☐	☐<br>☐<br>☐	☐<br>☐<br>☐	☐<br>☐<br>☐	☐<br>☐<br>☐
5.	Was the headache	on both sides:<br>right sided:<br>left sided:	☐<br>☐<br>☐	☐<br>☐<br>☐	☐<br>☐<br>☐	☐<br>☐<br>☐	☐<br>☐<br>☐	☐<br>☐<br>☐	☐<br>☐<br>☐
6.	Was the headache	pressing/tightening:<br>pulsating/throbbing:	☐<br>☐	☐<br>☐	☐<br>☐	☐<br>☐	☐<br>☐	☐<br>☐	☐<br>☐
7.	Was the headache - on average (see * below)	mild:<br>moderate:<br>severe:	☐<br>☐<br>☐	☐<br>☐<br>☐	☐<br>☐<br>☐	☐<br>☐<br>☐	☐<br>☐<br>☐	☐<br>☐<br>☐	☐<br>☐<br>☐
8.	Was the headache aggravated by physical activity, e.g. walking on stairs?	no:<br>yes:	☐<br>☐	☐<br>☐	☐<br>☐	☐<br>☐	☐<br>☐	☐<br>☐	☐<br>☐
9.	Did you suffer from nausea?	no:<br>mild:<br>moderate:<br>severe:	☐<br>☐<br>☐<br>☐	☐<br>☐<br>☐<br>☐	☐<br>☐<br>☐<br>☐	☐<br>☐<br>☐<br>☐	☐<br>☐<br>☐<br>☐	☐<br>☐<br>☐<br>☐	☐<br>☐<br>☐<br>☐
10.	Were you bothered by light?	no:<br>yes:	☐<br>☐	☐<br>☐	☐<br>☐	☐<br>☐	☐<br>☐	☐<br>☐	☐<br>☐
11.	Were you bothered by sounds?	no:<br>yes:	☐<br>☐	☐<br>☐	☐<br>☐	☐<br>☐	☐<br>☐	☐<br>☐	☐<br>☐
12.	Did anything trigger this headache attack	if yes, please specify:							
13.	Did you take any medicine for headache or for any other pain? (for each, please write name, amount and the time you took it)	name:							
		amount:							
		time:							
		name:							
		amount:							
		time:							
		name:							
		amount:							
		time:							

*) **Mild** = does not inhibit work or other activities.
Moderate = inhibits, but does not prohibit work or other activities.
Severe= prohibits work or other activities.

Supplementary Figure S2. Information box regarding Tension-type Headache Information box regarding Migraine

With tension-type headache at least 2/4 of the following must be fulfilled:

- Moderate/mild pain intensity
- Pain located on both side of the head.
- The pain is pressing/tightening.
- Not aggravated by routine physical activity

Besides there can be sound- or light sensitivity, but no nausea.

With migraine at least 2/4 of the following must be fulfilled:

- Moderate/severe pain intensity
- Pain is most prominent on one side of the head.
- The pain is pulsatile/pounding.
- Aggravation by routine physical activity

Besides there can be at least 1/2:

- Nausea
- Sound-or light sensitivity
